# *Trypanosoma cruzi* Promotes Transcriptomic Remodeling of the JAK/STAT Signaling and Cell Cycle Pathways in Myoblasts

**DOI:** 10.3389/fcimb.2020.00255

**Published:** 2020-06-17

**Authors:** Lindice M. Nisimura, Laura L. Coelho, Tatiana G. de Melo, Paloma de Carvalho Vieira, Pedro H. Victorino, Luciana R. Garzoni, David C. Spray, Dumitru A. Iacobas, Sanda Iacobas, Herbert B. Tanowitz, Daniel Adesse

**Affiliations:** ^1^Laboratório de Pesquisa em Apicomplexa, Instituto Carlos Chagas, Fundação Oswaldo Cruz, Curitiba, Brazil; ^2^Laboratório de Inovações em Terapias, Ensino e Bioprodutos, Instituto Oswaldo Cruz, Fundação Oswaldo Cruz, Rio de Janeiro, Brazil; ^3^Laboratório de Ultraestrutura Celular, Instituto Oswaldo Cruz, Fundação Oswaldo Cruz, Rio de Janeiro, Brazil; ^4^Laboratório de Biologia Estrutural, Instituto Oswaldo Cruz, Fundação Oswaldo Cruz, Rio de Janeiro, Brazil; ^5^Laboratório de Neurogênese, Instituto de Biofísica Carlos Chagas Filho, Universidade Federal do Rio de Janeiro, Rio de Janeiro, Brazil; ^6^Dominick P. Purpura Department of Neuroscience, Albert Einstein College of Medicine, New York, NY, United States; ^7^Personalized Genomics Laboratory, Center for Computational Systems Biology, Prairie View A&M University, Prairie View, TX, United States; ^8^Department of Pathology, New York Medical College, Valhalla, NY, United States; ^9^Department of Pathology, Albert Einstein College of Medicine, New York, NY, United States

**Keywords:** Chagas disease, myoblasts, cell cycle, JAK-STAT pathway, *Clcf1*, *Ywhaq*

## Abstract

Chagas disease is responsible for more than 10,000 deaths per year and about 6 to 7 million infected people worldwide. In its chronic stage, patients can develop mega-colon, mega-esophagus, and cardiomyopathy. Differences in clinical outcomes may be determined, in part, by the genetic background of the parasite that causes Chagas disease. *Trypanosoma cruzi* has a high genetic diversity, and each group of strains may elicit specific pathological responses in the host. Conflicting results have been reported in studies using various combinations of mammalian host—*T. cruzi* strains. We previously profiled the transcriptomic signatures resulting from infection of L6E9 rat myoblasts with four reference strains of *T. cruzi* (Brazil, CL, Y, and Tulahuen). The four strains induced similar overall gene expression alterations in the myoblasts, although only 21 genes were equally affected by all strains. *Cardiotrophin-like cytokine factor 1* (*Clcf1*) was one of the genes found to be consistently upregulated by the infection with all four strains of *T. cruzi*. This cytokine is a member of the interleukin-6 family that binds to glycoprotein 130 receptor and activates the JAK/STAT signaling pathway, which may lead to muscle cell hypertrophy. Another commonly upregulated gene was tyrosine 3-monooxygenase/tryptophan 5-monooxygenase activation protein theta (*Ywhaq*, 14-3-3 protein Θ), present in the Cell Cycle Pathway. In the present work, we reanalyzed our previous microarray dataset, aiming at understanding in more details the transcriptomic impact that each strain has on JAK/STAT signaling and Cell Cycle pathways. Using Pearson correlation analysis between the expression levels of gene pairs in biological replicas from each pathway, we determined the coordination between such pairs in each experimental condition and the predicted protein interactions between the significantly altered genes by each strain. We found that although these highlighted genes were similarly affected by all four strains, the downstream genes or their interaction partners were not necessarily equally affected, thus reinforcing the idea of the role of parasite background on host cell transcriptome. These new analyses provide further evidence to the mechanistic understanding of how distinct *T. cruzi* strains lead to diverse remodeling of host cell transcriptome.

## Introduction

Chagas disease (CD) is caused by the protozoan *Trypanosoma cruzi* and affects about 6 to 7 million people worldwide (WHO, 2019). The cardiac form of CD (Mukherjee et al., 2003; Goldenberg et al., 2009; Soares et al., 2010; Adesse et al., 2011) is the main clinical manifestation, which can be observed in more than 30% of chronically infected people, whereas another 10% develop digestive, neurological, or mixed alterations (Rassi et al., 2010, 2012; WHO, 2019). These diverse presentations might in part be explained by genetic differences between strains of *T. cruzi*, which have been classified into six discrete typing units (DTUs) (Andrade and Magalhaes, 1997; Zingales et al., 2009). This classification is based on distinct ecological, epidemiological, natural, and experimental infection features of the parasite, but clinical manifestations are not strictly associated with the specific *T. cruzi* DTUs (Zingales et al., 2012). In order to understand the variations in CD severity and tissue specificity, there is a need to identify key molecular biomarkers and to correlate the gene expression profiles of *T. cruzi* strains with CD pathogenicity.

We previously compared gene expression profiles in a rat myoblast cell line (L6E9) infected with four different strains of *T. cruzi* (Brazil, Y, CL, and Tulahuen) (Adesse et al., 2010a). That study identified up regulation of *cardiotrophin-like cytokine factor 1* (*Clcf1*) by all four strains of *T. cruzi. Clcf1* belongs to the interleukin (IL)-6 family of cytokines that have the glycoprotein gp130 as a common signal-transducing receptor and is involved in cell differentiation, survival, apoptosis, and proliferation through activation of Janus kinase (JAK). JAKs in turn, activate signal transducer and activator of transcription (STAT) factors (Gorshkova et al., 2016). CLCF1 has been reported to induce hypertrophy and survival of cardiomyocytes *in vitro* (Sheng et al., 1996; Latchman, 1999) through gp130 and STAT3 pathway activation (Kunisada et al., 1998). Plasma levels of CLCF1 are correlated with severity of hypertrophy in patients with hypertrophic cardiomyopathy or hypertension (Monserrat et al., 2011; Song et al., 2014). In acute experimental CD, rats infected with *T. cruzi* (Sylvio X10/7 strain, TcI) revealed cardiac overexpression of CLCF1 and gp130 (Chandrasekar et al., 1998). These data could explain in part why the predominant DTUs in our previous study (TcI and TcII) are associated with cardiac manifestation of CD.

STAT proteins include STAT1–4, –5a, –5b, and –6 and have been shown to play an important role in cytokine signaling. These proteins are tyrosine phosphorylated by JAKs following the binding of cytokine to its receptor. Upon tyrosine phosphorylation, STAT proteins form homodimers or heterodimers and rapidly translocate to the nucleus and induce gene expression. Recent evidence has demonstrated the necessity of STAT3 in cell growth and transformation (Zong et al., 2000; Ponce et al., 2013; Stahl et al., 2013). The JAK/STAT pathway is involved in cell cycle regulation, and it has been shown that myoblast proliferation involves this pathway (Sorensen et al., 2018; Steyn et al., 2019). JAK1 and STAT1 induce cell proliferation and reduce myogenic differentiation (Sun et al., 2007). Additionally, phosphorylation of JAK2–STAT5 has been shown to protect skeletal muscle in acute aerobic exercise (Consitt et al., 2008).

Regarding *T. cruzi* infection, STAT3 phosphorylation induces cardiomyocyte protection against apoptosis through increased expression of anti-apoptotic factor Bcl-2 (Ponce et al., 2012). Cell cycle was modulated distinctly in cells infected with the Dm28c (type I) and the Y and CL-Brener *T. cruzi* stocks (type II), and there were different levels of apoptosis induction by each strain. Moreover, *T. cruzi* infection provoked variable apoptosis rates in distinct host cell types (cardiomyocytes, fibroblasts, and macrophages) (de Souza et al., 2003).

In this context, transcriptomic analyses may be expected to elucidate associations between the DTUs and prediction of the pathogenesis of *T. cruzi* strains. In the present study, we focus on determination of the transcriptomic impact of each strain on JAK/STAT signaling and cell cycle pathways. Novel bioinformatics tools were used to reanalyze the data generated by microarray analysis of L6E9 cells infected with four distinct strains of *T. cruzi* (Brazil, CL, Y, and Tulahuen).

## Methods

### Experimental Design

For microarrays analyses, rat skeletal myoblast L6E9 were used as described in Adesse et al. (2010a). Before reaching confluency, cells were dissociated with trypsin/EDTA in phosphate-buffered saline (PBS) and plated for experiments. Trypomastigotes of *Trypanosoma cruzi* were isolated from supernatants of infected Vero cells and used at a multiplicity of infection (MOI) of 10. Twenty-four hours after infection, cultures were washed twice with Ringer's saline solution and fresh supplemented medium was added. Medium was replaced daily and cultures were kept up to 72 h post infection.

### Microarray

Microarray data were obtained from our previous publication (Adesse et al., 2010a), and the experimental design and procedures are described in brief as follows. Cell culture dishes containing the L6E9 rat myoblast cell line were infected with trypomastigote forms of *T. cruzi* (Y, CL Brener, Tulahuen, and Brazil strains) (see Adesse et al., 2010a, for details). Total RNA was harvested 72 h post infection using TRIzol reagent (Invitrogen, Carlsbad, CA), following the protocol indicated by the manufacturer. Microarray analysis was performed using the protocol optimized in our laboratory according to the standards of the Microarray Gene Expression Data Society. Differently labeled RNA samples from biological replicas of control (uninfected cells cultured for the same duration) or infected with one strain at a time were co-hybridized (“multiple yellow” strategy) with rat oligonucleotide arrays printed by Duke University. The abundance of host cell transcripts was considered as significantly altered after infection if the absolute fold change was >1.5-fold and the *P*-value of the heteroscedastic *t*-test (two-sample, unequal variance) was >0.05. Experimental details and raw and processed expression data have been deposited and are publically available at https://www.ncbi.nlm.nih.gov/geo/query/acc.cgi?acc=GSE18175.

### Expression Coordination Analysis

As previously described (Iacobas et al., 2008a), the gene networks in uninfected control L6E9 myocytes and those infected with each of the four strains (Brazil, CL, Tulahuen, or Y strain) were established by calculating pairwise Pearson correlation coefficients of the (log_2_) expression levels of each pair of pathway genes in the biological replicas. Two genes were considered as synergistically expressed if their expression levels increased and decreased together (positive covariance) in a set of similar samples or as antagonistically expressed (negative covariance) when they manifest opposite tendencies and as independently expressed when their expressions are not correlated (close to zero covariance). In the case of four biological replicas, the (*p* < 0.05) cut-off for synergism is a pairwise Pearson correlation coefficient ρ > 0.90, for antagonism ρ < −0.90, and for independence |ρ| < 0.05. To illustrate, Supplementary Figure 1 presents examples of synergistically (*Antxr1*), antagonistically (*Dus3l*), and independently (*Golim4*) expressed genes with *Clcf1* in control L6E9 rat myoblasts.

### “See-Saw” Partners of Key Genes

For each gene of interest and each experimental condition, we determined the coordination profile, defined as the set of Pearson correlation coefficients between the expression levels within biological replicas of that gene and each other gene. We then identified the gene pairs with very similar or opposite coordination profiles in each condition, termed “see-saw” partners, both in recognition of the appearance of the graphs and to denote the strength of their synergistic and antagonistic relationships (Iacobas et al., 2007a,b, 2008a,b; Spray and Iacobas, 2007).

### Pathway Analysis

On the basis of our initial analyses of genes whose expression was altered by infection with different *T. cruzi* strains, we selected the following gene pathways for further analysis using the Kyoto Encyclopedia of Genes and Genomes (KEGG): a) JAK/STAT Signaling Pathway (http://www.kegg.jp/kegg-bin/show_pathway?org_name=rno&mapno=04630&mapscale=1.0&show_description=hide) and b) Cell Cycle Pathway (http://www.kegg.jp/kegg-bin/show_pathway?org_name=rno&mapno=04110&mapscale=1.0&show_description=hide).

## Results

### Differential Alterations in Predicted Protein–Protein Interactions (PPI) in L6E9 Myoblasts Infected With Distinct *Trypanosoma cruzi* Strains

In our previous paper (Adesse et al., 2010a), randomly selected genes were presented among those that were significantly altered by each strain of *Trypanosoma cruzi*. We now listed the 30 genes most downregulated and upregulated genes by each strain (Tables 1–8). Whereas the fold changes of the most strongly downregulated genes were similar (about −4- to −2-fold), fold changes of upregulated genes were as high as 13- to 54-fold in the various *T. cruzi* strains. The Y strain was the least disruptive for the transcriptome because fold changes of the 30 most strongly upregulated genes were notably lower than for the other strains.

**Table 1:** Top 30 upregulated genes by Brazil strain-infected L6E9 cells at 72 hpi.

**Gene name**	**Gene symbol**	**Fold change**
Erythroid spectrin beta	LOC314251	35.5
Transmembrane protease, serine 11d	Tmprss11d	20.9
4-Hydroxyphenylpyruvic acid dioxygenase	Hpd	11.9
IQ motif and Sec7 domain 3	Iqsec3	10.2
DNA-damage inducible transcript 3	Ddit3	9.3
One cut domain, family member 1	Onecut1	9.3
Olfactory receptor 1751 (predicted)	Olr1751_predicted	9.3
Olfactory receptor 135 (predicted)	Olr135_predicted	8.9
Cut-like 1 (*Drosophila*)	Cutl1	
Proprotein convertase subtilisin/kexin type 7	Pcsk7	6.9
Olfactory receptor 3 (predicted)	Olr3_predicted	5.9
Amiloride binding protein 1 (amine oxidase, copper-containing)	Abp1	5.7
Arylacetamide deacetylase (esterase)	Aadac	5.5
EGF-like domain 7	Egfl7	5.5
Similar to 60S ribosomal protein L29 (P23) (predicted)	RGD1566397_predicted	5.5
Similar to RIKEN cDNA 0610012D17 (predicted)	RGD1564702_predicted	5.4
Similar to 60S ribosomal protein L23a	LOC291686	5.2
Potassium channel, subfamily K, member 2	Kcnk2	5.1
Cdc2-related kinase, arginine/serine-rich	Crkrs	5.1
PITPNM family member 3 (predicted)	Pitpnm3_predicted	5.0
Ras association (RalGDS/AF-6) domain family 3 (predicted)	Rassf3_predicted	5.0
Similar to DIP13 beta (predicted)	RGD1563028_predicted	4.9
Similar to ribosomal protein L13 (predicted)	RGD1563194_predicted	4.6
Similar to BTB and CNC homology 1, basic leucine zipper transcription factor 2 (predicted)	RGD1562865_predicted	4.5
Elastin	Eln	4.4
RT1 class I, CE15	RT1-CE15	4.4
Adaptor-related protein complex 3, mu 2 subunit	Ap3m2	4.4
Rhesus blood group-associated C glycoprotein	Rhcg	4.3
FYVE and coiled-coil domain containing 1 (predicted)	Fyco1_predicted	4.3

**Table 2:** Top 30 downregulated genes by Brazil strain-infected L6E9 cells at 72 hpi.

**Gene name**	**Gene symbol**	**Fold change**
Solute carrier family 39 (metal ion transporter), member 6	Slc39a6	−2.8
Glycoprotein (transmembrane) nmb	Gpnmb	−2.5
Tropomyosin 4	Tpm4	−2.5
Parkinson disease (autosomal recessive, early onset) 7	Park7	−2.4
ATP-binding cassette, sub-family E (OABP), member 1	Abce1	−2.4
Keratin 25D	Krt25d	−2.4
Proteasome (prosome, macropain) 28 subunit, beta	Psme2	−2.3
Similar to hypothetical protein MGC40499 (predicted)	RGD1307636_predicted	−2.3
Milk fat globule-EGF factor 8 protein	Mfge8	−2.3
Macoilin	LOC313618	−2.3
NADPH oxidase 3	Nox3	−2.3
Similar to RIKEN cDNA 9630046K23	RGD1306248	−2.3
Xylulokinase homolog (*Haemophilus influenzae*)	Xylb	−2.2
Similar to A disintegrin-like and metalloprotease (reprolysin type) with thrombospondin type 1 motif, 2 (predicted)	RGD1565950_predicted	−2.2
COP9 (constitutive photomorphogenic) homolog, subunit 4 (Arabidopsis thaliana)	Cops4	−2.1
Ankyrin repeat domain 1 (cardiac muscle)	Ankrd1	−2.1
Gasdermin domain containing 1 (predicted)	Gsdmdc1_predicted	−2.1
Similar to mKIAA1011 protein	LOC366669	−2.1
Similar to RIKEN cDNA 1500016L11 (predicted)	RGD1305050_predicted	−2.1
Ribosomal protein L28	Rpl28	−2.1
Thymoma viral proto-oncogene 1	Akt1	−2.1
SEC24 related gene family, member A (*Saccharomyces cerevisiae*) (predicted)	Sec24a_predicted	−2.0
Adaptor protein complex AP-2, alpha 2 subunit	Ap2a2	−2.0
Guanosine diphosphate dissociation inhibitor 1	Gdi1	−2.0
Similar to hypothetical protein MGC25461 (predicted)	RGD1306717_predicted	−2.0
Matrix metallopeptidase 14 (membrane-inserted)	Mmp14	−2.0
Rho GTPase activating protein 27	Arhgap27	−2.0
Voltage-dependent anion channel 2	Vdac2	−1.9
C1q and tumor necrosis factor-related protein 1	C1qtnf1	−1.9

**Table 3:** Top 30 upregulated genes by CL strain-infected L6E9 cells at 72 hpi.

**Gene name**	**Gene symbol**	**Fold change**
Similar to protein phosphatase 1, regulatory (inhibitory) subunit 1C; thymocyte ARPP; DNA segment, Chr 9, Brigham and Women's Genetics 1012 expressed	RGD1307215	43.5
Calcium/calmodulin-dependent protein kinase I gamma	Camk1g	29.6
Chemokine (C-X-C motif) ligand 10	Cxcl10	20.8
5-Methyltetrahydrofolate-homocysteine methyltransferase	Mtr	16.5
Olfactory receptor 813 (predicted)	Olr813_predicted	9.9
Transmembrane protease, serine 11d	Tmprss11d	9.6
ATP-binding cassette, sub-family G (WHITE), member 3	Abcg3	7.7
Nuclear receptor subfamily 4, group A, member 1	Nr4a1	6.2
Peptidyl arginine deiminase, type I	Padi1	4.3
Pericentriolar material 1	Pcm1	4.3
ATPase, H+ transporting, lysosomal V0 subunit A isoform 4 (predicted)	Atp6v0a4_predicted	3.7
Claspin homolog (*Xenopus laevis*) (predicted)	Clspn_predicted	3.6
Mitochondrial trans-2-enoyl-CoA reductase	Mecr	3.5
Checkpoint kinase 1 homolog (*Schizosaccharomyces pombe*)	Chek1	3.5
Similar to RIKEN cDNA 6530401L14 gene	RGD1309107	3.4
Radial spokehead-like 2 (predicted)	Rshl2_predicted	3.4
UDP-glucose ceramide glucosyltransferase-like 1	Ugcgl1	3.4
Glutamate receptor, ionotropic, *N*-methyl d-aspartate 2B	Grin2b	3.4
Similar to RIKEN cDNA 4921513E08 (predicted)	RGD1305153_predicted	3.2
Similar to RIKEN cDNA 2700097O09 (predicted)	RGD1304624_predicted	3.2
Similar to RIKEN cDNA A530088I07 gene	LOC311984	3.1
Leucine rich repeat protein 3, neuronal	Lrrn3	3.1
Erythroid spectrin beta	LOC314251	3.1
DNA-damage inducible transcript 3	Ddit3	3.1
Peroxisomal biogenesis factor 11c (predicted)	Pex11c_predicted	3.1
Potassium channel tetramerization domain containing 1	Kctd1	3.1
Similar to hypothetical protein FLJ31846 (predicted)	RGD1306118_predicted	3.1
ATH1, acid trehalase-like 1 (yeast) (predicted)	Athl1_predicted	3.0
Interleukin-21 receptor	Il21r	3.0
Similar to pseudouridylate synthase-like 1	LOC362681	3.0

**Table 4:** Top 30 downregulated genes by CL strain-infected L6E9 cells at 72 hpi.

**Gene name**	**Gene symbol**	**Fold change**
V-maf musculoaponeurotic fibrosarcoma oncogene homolog (avian)	Maf	−3.1
Glycoprotein (transmembrane) nmb	Gpnmb	−2.8
T-cell immunomodulatory protein	Cda08	−2.6
Succinate-Coenzyme A ligase, ADP-forming, beta subunit (predicted)	Sucla2_predicted	−2.6
OMA1 homolog, zinc metallopeptidase (*Saccharomyces cerevisiae*) (predicted)	Oma1_predicted	−2.4
Similar to CG9996-PA	LOC300173	−2.3
Regenerating islet-derived 1	Reg1	−2.3
Phosphotriesterase related	Pter	−2.2
Similar to myosin, light polypeptide 6, alkali, smooth muscle and non-muscle (predicted)	RGD1559821_predicted	−2.1
Syndecan 1	Sdc1	−2.1
Pyruvate dehydrogenase kinase, isoenzyme 2	Pdk2	−2.0
Aminoadipate-semialdehyde dehydrogenase-phosphopantetheinyl transferase (predicted)	Aasdhppt_predicted	−2.0
Matrix metallopeptidase 14 (membrane-inserted)	Mmp14	−1.9
Solute carrier family 27 (fatty acid transporter), member 1	Slc27a1	−1.8
Similar to phosphatidylglycerophosphate synthase (predicted)	RGD1305052_predicted	−1.8
UDP-Gal:betaGlcNAc beta 1,4-galactosyltransferase, polypeptide 5 (predicted)	B4galt5_predicted	−1.8
Spondin 2, extracellular matrix protein	Spon2	−1.8
Spastic paraplegia 21 homolog (human)	Spg21	−1.8
C1q and tumor necrosis factor-related protein 1	C1qtnf1	−1.8
Neuropathy target esterase like 1	Ntel1	−1.8
Similar to 60S ribosomal protein L35 (predicted)	RGD1562863_predicted	−1.8
Carnitine palmitoyltransferase 1a, liver	Cpt1a	−1.8
Gametogenetin-binding protein 1	Ggnbp1	−1.8
F-box and leucine-rich repeat protein 3	Fbxl3	−1.7
Homer homolog 3 (*Drosophila*)	Homer3	−1.7
Phospholipase C, gamma 1	Plcg1	−1.7
DEAH (Asp-Glu-Ala-His) box polypeptide 16	Dhx16	−1.7
Similar to C530044N13Rik protein	RGD1306568	−1.7
Similar to CG4768-PA (predicted)	RGD1309748_predicted	−1.7
UDP-*N*-acetylglucosamine pyrophosphorylase 1-like 1 (predicted)	Uap1l1_predicted	−1.7

**Table 5:** Top 30 upregulated genes by Tulahuen strain-infected L6E9 cells at 72 hpi.

**Gene name**	**Gene symbol**	**Fold change**
Calcium/calmodulin-dependent protein kinase I gamma	Camk1g	57.6
Chemokine (C-X-C motif) ligand 10	Cxcl10	35.9
Similar to Set alpha isoform	LOC317165	20.2
Erythroid spectrin beta	LOC314251	20.2
Similar to protein phosphatase 1, regulatory (inhibitory) subunit 1C; thymocyte ARPP; DNA segment, Chr 9, Brigham and Women's Genetics 1012 expressed	RGD1307215	19.1
Vacuolar protein sorting 37C (yeast) (predicted)	Vps37c_predicted	14.5
5-Methyltetrahydrofolate-homocysteine methyltransferase	Mtr	13.7
ATP-binding cassette, sub-family G (WHITE), member 3	Abcg3	12.2
Similar to BCL6 co-repressor-like 1 (predicted)	RGD1566108_predicted	10.1
Transmembrane protease, serine 11d	Tmprss11d	9.7
Interleukin-21 (predicted)	Il21_predicted	9.4
Matrix metallopeptidase 15 (predicted)	Mmp15_predicted	7.8
Activin A receptor type II-like 1	Acvrl1	7.6
Olfactory receptor 3 (predicted)	Olr3_predicted	7.4
Oncomodulin	Ocm	7.1
Olfactory receptor 889 (predicted)	Olr889_predicted	6.9
Seminal vesicle secretion 1	Svs1	6.7
Olfactory receptor 859 (predicted)	Olr859_predicted	6.7
Olfactory receptor 155 (predicted)	Olr155_predicted	6.1
Arylacetamide deacetylase (esterase)	Aadac	5.8
Homeo box, msh-like 3	Msx3	5.7
4-hydroxyphenylpyruvic acid dioxygenase	Hpd	5.5
Beta-1,3-glucuronyltransferase 1 (glucuronosyltransferase P)	B3gat1	5.4
CD4 antigen	Cd4	5.4
Cut-like 1 (*Drosophila*)	Cutl1	5.4
BMP and activin membrane-bound inhibitor, homolog (*Xenopus laevis*)	Bambi	5.3
Similar to hypothetical protein FLJ31846 (predicted)	RGD1306118_predicted	5.2
Slit homolog 3 (*Drosophila*)	Slit3	5.2
Eyes absent 2 homolog (*Drosophila*)	Eya2	5.1
PDZ domain containing 6 (predicted)	Pdzk6_predicted	5.0

**Table 6:** Top 30 downregulated genes by Tulahuen strain-infected L6E9 cells at 72 hpi.

**Gene name**	**Gene symbol**	**Fold change**
Procollagen, type XVI, alpha 1	Col16a1	−4.7
Solute carrier family 16 (monocarboxylic acid transporters), member 1	Slc16a1	−3.8
Inositol 1,4,5-triphosphate receptor 3	Itpr3	−3.7
Matrix metallopeptidase 11	Mmp11	−3.5
Exocyst complex component 7	Exoc7	−3.4
V-abl Abelson murine leukemia viral oncogene homolog 1	Abl1	−3.3
Dermatopontin (predicted)	Dpt_predicted	−3.2
Guanosine monophosphate reductase 2	Gmpr2	−3.2
Discoidin domain receptor family, member 1	Ddr1	−3.2
Tetraspanin 5	Tspan5	−3.2
Olfactory receptor 865 (predicted)	Olr865_predicted	−3.1
Similar to hypothetical protein (predicted)	RGD1561605_predicted	−3.1
Protein phosphatase 1 (formerly 2C)-like (predicted)	Ppm1l_predicted	−3.0
Bcl2 modifying factor	Bmf	−2.9
IAP promoted placental gene (predicted)	Ipp_predicted	−2.9
AE binding protein 1 (predicted)	Aebp1_predicted	−2.9
Ectonucleoside triphosphate diphosphohydrolase 1	Entpd1	−2.9
Troponin T2, cardiac	Tnnt2	−2.9
Spondin 2, extracellular matrix protein	Spon2	−2.9
Phospholipase D2	Pld2	−2.9
Signal recognition particle receptor (“docking protein”)	Srpr	−2.9
Similar to late endosomal/lysosomal Mp1 interacting protein (p14) (predicted)	RGD1562501_predicted	−2.9
Tripeptidyl peptidase I	Tpp1	−2.8
A disintegrin and metallopeptidase domain 33 (predicted)	Adam33_predicted	−2.8
Similar to RIKEN cDNA 2010012O05 (predicted)	RGD1311783_predicted	−2.8
F-box protein 38 (predicted)	Fbxo38_predicted	−2.8
NADH dehydrogenase (ubiquinone) 1 beta subcomplex 3 (predicted)	Ndufb3_predicted	−2.8
RNA binding motif protein 4 (predicted)	Rbm4_predicted	−2.8
RAB8B, member RAS oncogene family	Rab8b	−2.8
Spastin (predicted)	Spast_predicted	−2.8

**Table 7:** Top 30 upregulated genes by Y strain-infected L6E9 cells at 72 hpi.

**Gene name**	**Gene symbol**	**Fold change**
Oncomodulin	Ocm	13.5
Syntaxin binding protein 5 (tomosyn)	Stxbp5	11.8
Similar to hypothetical protein FLJ31846 (predicted)	RGD1306118_predicted	8.9
ATP-binding cassette, sub-family G (WHITE), member 3	Abcg3	8.4
Ankyrin repeat and SOCS box-containing protein 3 (predicted)	Asb3_predicted	6.3
Slit homolog 3 (*Drosophila*)	Slit3	5.5
Elastin	Eln	5.1
Zinc finger protein 13 (predicted)	Zfp13_predicted	4.6
BMP and activin membrane-bound inhibitor, homolog (*Xenopus laevis*)	Bambi	4.5
FYVE and coiled-coil domain containing 1 (predicted)	Fyco1_predicted	4.4
Interleukin-21 (predicted)	Il21_predicted	4.4
Galactose mutarotase	Galm	4.3
Presenilin 2	Psen2	3.8
Pericentriolar material 1	Pcm1	3.7
Guanylate cyclase activator 2a (guanylin)	Guca2a	3.5
PDZ domain containing 6 (predicted)	Pdzk6_predicted	3.3
5-Methyltetrahydrofolate-homocysteine methyltransferase	Mtr	3.2
Similar to transcription factor (p38 interacting protein)	RGD1307812	3.1
UDP-glucose ceramide glucosyltransferase-like 1	Ugcgl1	3.1
Similar to protein phosphatase 1, regulatory (inhibitory) subunit 1C; thymocyte ARPP; DNA segment, Chr 9, Brigham and Women's Genetics 1012 expressed	RGD1307215	3.0
Eyes absent 2 homolog (*Drosophila*)	Eya2	3.0
Pregnancy-specific beta 1-glycoprotein	LOC292668	2.9
Ets variant gene 4 (E1A enhancer binding protein, E1AF) (predicted)	Etv4_predicted	2.9
Transferrin receptor	Tfrc	2.9
Similar to RIKEN cDNA 2700097O09 (predicted)	RGD1304624_predicted	2.9
Jumonji domain containing 3 (predicted)	Jmjd3_predicted	2.9
Similar to hypothetical protein FLJ10342 (predicted)	RGD1307791_predicted	2.8
RNA pseudouridylate synthase domain containing 2 (predicted)	Rpusd2_predicted	2.8
Transmembrane protein 12	Tmem12	2.8
Oxidoreductase NAD-binding domain containing 1 (predicted)	Oxnad1_predicted	2.7

**Table 8:** Top 30 downregulated genes by Y strain-infected L6E9 cells at 72 hpi.

**Gene name**	**Gene symbol**	**Fold change**
Actin, alpha 1, skeletal muscle	Acta1	−4.8
Similar to cDNA sequence BC019755 (predicted)	RGD1306601_predicted	−3.3
Cytochrome P450, family 2, subfamily d, polypeptide 22	Cyp2d22	−2.9
Cadherin 15	Cdh15	−2.9
Cytochrome P450, family 26, subfamily b, polypeptide 1	Cyp26b1	−2.8
Olfactory receptor 865 (predicted)	Olr865_predicted	−2.7
Ankyrin repeat domain 1 (cardiac muscle)	Ankrd1	−2.7
Similar to nuclease sensitive element binding protein 1	LOC367118	−2.7
Acyl-CoA synthetase long-chain family member 3	Acsl3	−2.6
Dispatched homolog 1 (*Drosophila*) (predicted)	Disp1_predicted	−2.6
Carbonic anhydrase 3	Ca3	−2.6
Inhibitor of growth family, member 3	Ing3	−2.6
C-fos-induced growth factor	Figf	−2.6
Naked cuticle 2 homolog (*Drosophila*) (predicted)	Nkd2_predicted	−2.5
Inhibitor of DNA binding 4	Id4	−2.4
Cytidine 5′-triphosphate synthase (predicted)	Ctps_predicted	−2.4
Similar to Eso3 protein (predicted)	RGD1562476_predicted	−2.3
Cysteine and glycine-rich protein 2	Csrp2	−2.3
RAC/CDC42 exchange factor	Geft	−2,26947
Similar to DNA segment, Chr 8, ERATO Doi 82, expressed (predicted)	RGD1311793_predicted	−2.3
Stanniocalcin 2	Stc2	−2.2
Family with sequence similarity 3, member C	Fam3c	−2.2
Cohen syndrome homolog 1 (predicted)	Cohh1_predicted	−2.2
Guanosine monophosphate reductase	Gmpr	−2.2
Similar to RIKEN cDNA 9630046K23	RGD1306248	−2.2
Unc-51-like kinase 1	Ulk1	−2.2
DnaJ (Hsp40) homolog, subfamily A, member 4	Dnaja4	−2.2
Ephrin B1	Efnb1	−2.1
Dermatopontin (predicted)	Dpt_predicted	−2.1
Testis expressed gene 264 homolog (mouse)	Tex264	−2.1

**Figure 1:**
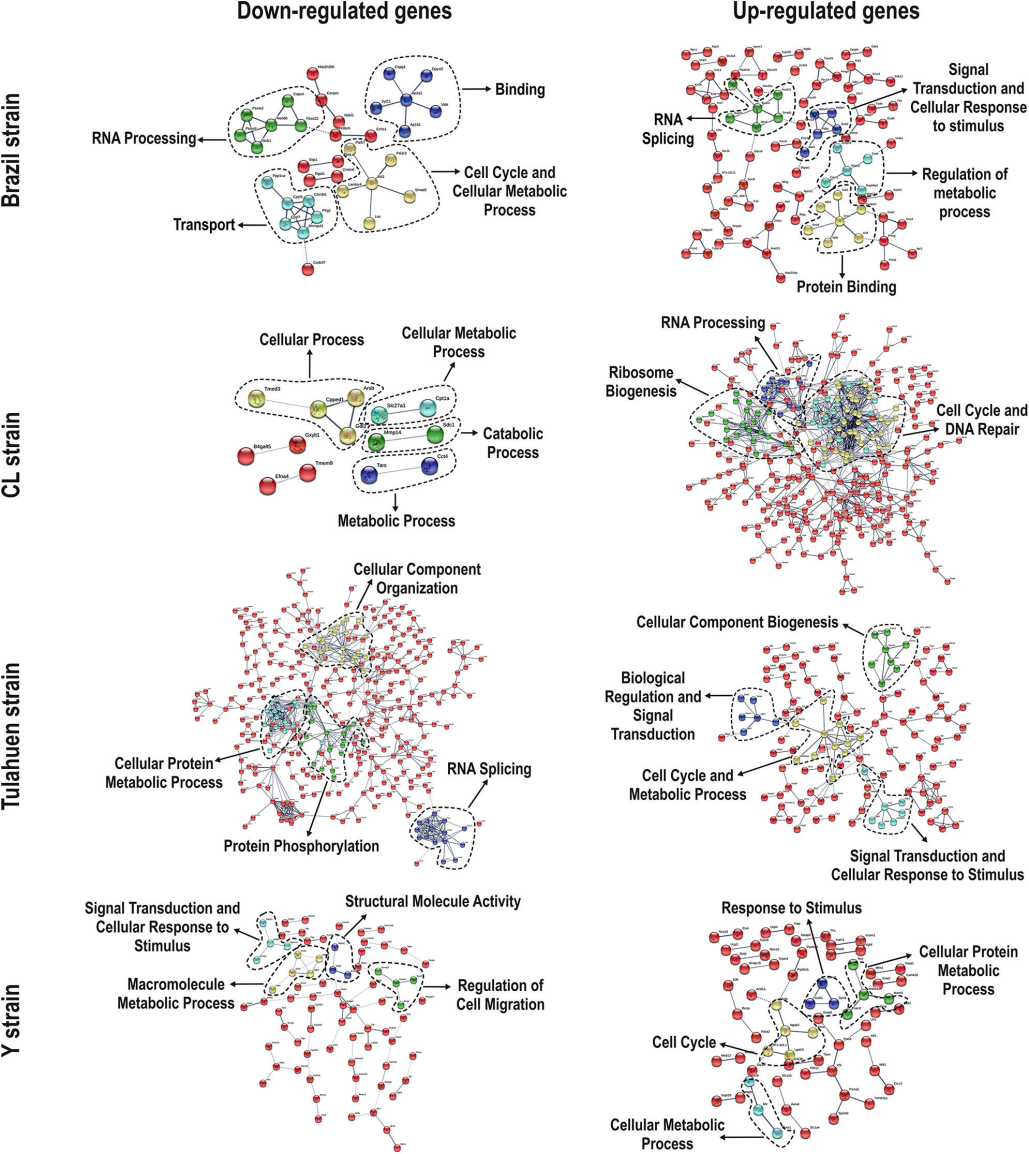
Protein–protein interaction (PPI) networks revealed by upregulated and downregulated genes after infection with each *Trypanosoma cruzi* strain. Among the downregulated (**left** panels) and upregulated genes (**right** panels), we obtained/assembled PPIs using STRING software, with a confidence cut-off that ranged from 0.4 to 0.7. Nodes labeled with the encoding gene symbol indicate proteins, and the lines represent the corresponding interactions. The confidence score of each interaction is mapped to the line thickness (the thicker the line, the more evidence to support the interaction). The network was then enriched according to a gene ontology database. Subsequent analysis with K-means algorithm predicted nodes of interacting proteins (highlighted with the dashed lines), and such nodes were determined with Pathvisio assigning their molecular functions.

We used the STRING platform to predict protein–protein interaction (PPI) networks and subsequently applied K-means algorithm to determine clusters of genes with a similar expression profile (Figure 1). The clusters that were generated were then analyzed by Pathvisio software (Kutmon et al., 2015), in order to determine their molecular function; these clusters and their associated functions are shown in Figure 1. Among 111 downregulated genes, the Brazil strain affected the “RNA processing,” “binding,” “transport,” and “cell cycle and cellular metabolic process” pathways, and 377 upregulated genes involved “RNA splicing,” “signal transduction and cellular response to stimulus,” “regulation of metabolic process,” and “protein binding.” The CL strain downregulated the expression of 53 genes, but only nine interactions were found among 14 regulated genes, belonging to the “cellular process,” “cellular metabolic process,” “catabolic process,” and “metabolic process” categories. Regarding the 764 genes of the L6E9 cells that were upregulated by the CL, we found a total of 884 interactions that could be grouped into three main biological processes: “ribosome biogenesis,” “RNA processing,” and “cell cycle and DNA repair.” By contrast, analysis of the Tulahuen-infected samples revealed that most of the 1,144 differentially expressed genes (DEGs) were downregulated (761 genes), which formed 494 interactions. Such interactions were grouped into four main clusters: “cellular component organization,” “RNA splicing,” “protein phosphorylation,” and “cellular protein metabolic process,” each with 22, 15, 22, and 18 genes, respectively. The remaining 383 DEGs by the Tulahuen strain were all upregulated and generated 146 interactions with four main biological processes: “cellular component biogenesis,” “biological regulation and signal transduction,” “signal transduction and cellular response to stimulus,” and “cell cycle and metabolic process” (Figure 1). Finally, we analyzed the DEGs from the Y strain-infected samples. We found a total of 68 and 44 predicted interactions when looking at 150 downregulated and 276 upregulated genes, respectively. Among the biological processes found among the downregulated network, we found “signal transduction and cellular response to stimulus,” “structural molecule activity,” “regulation of cell migration,” and “macromolecule metabolic process”; and in the upregulated genes, the “cell cycle,” “response to stimulus,” “cellular protein process,” and “cellular metabolic process” were found (Figure 1).

### *Trypanosoma cruzi* Strains Differentially Alter the Expression of JAK/STAT Signaling Pathway Members

**Figure 2:**
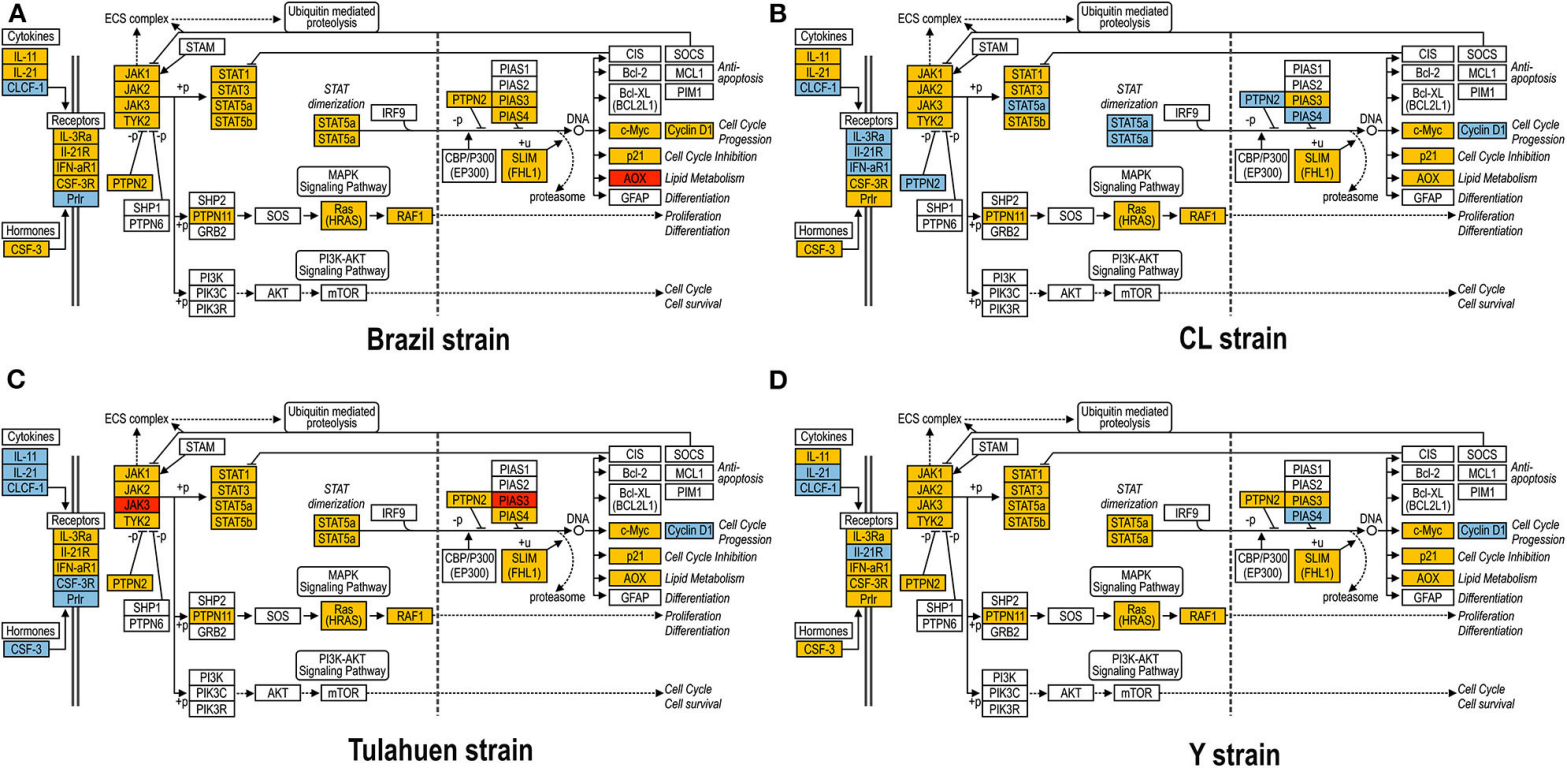
Kyoto Encyclopedia of Genes and Genomes (KEGG) analysis of the JAK/STAT Pathway in *Trypanosoma cruzi*-infected myoblasts. The JAK/STAT Pathway obtained from the KEGG platform was used as template to highlight the effect of Brazil **(A)**, CL **(B)**, Tulahuen **(C)**, and Y **(D)** strain in L6E9 rat myoblasts. Blue boxes indicate significant upregulation (>1.5-fold), and red boxes indicate significant downregulation (<-1.5-fold). Yellow boxes indicate genes that showed no significant alteration in infected vs. control cultures, whereas white boxes indicate genes that were absent in the analysis (i.e., those that for any reason did not match the exclusion criteria; e.g., a positive signal in all four biological replicas of each group). Extracellular ligands such as IL-6 family of cytokines bind to membrane receptors, including IL-3R and IL-21R, which in turn activate members of the JAK/STAT pathway or, alternatively, of the MAPK signaling pathway. STATs translocate to cell nucleus and activate transcription of genes that can activate cell cycle, apoptosis, or cell differentiation.

As previously described, all four strains of *T. cruzi* induced upregulation of the *Clcf1* transcript in infected rat myoblasts by 2.2-, 2.3-, 1.8-, and 2.3-fold by the Brazil, CL, Tulahuen, and Y strains, respectively. Because this cytokine is one of the known activators of the JAK/STAT signaling pathway, we investigated whether other genes in such pathway might be altered by all four strains, which would validate JAK/STAT activation as a hallmark of *T. cruzi* infection. Using KEGG pathway database, we highlighted which genes were significantly upregulated or downregulated by each parasite strain (Figure 2). We found that the CL strain, an isolate from the southern part of Brazil (TcVI), had the highest impact on JAK/STAT signaling pathway, inducing alteration in 37% of the 30 genes detected by the arrays. The Tulahuen strain, a Chilean isolate (also TcVI), altered 30% of the genes, whereas the Brazil and Y strains showed fewer pathway elements altered (3/30:10% and 5/30: 17%, respectively). In initial steps of this signaling cascade, the Brazil and CL strains upregulated only *Clcf1* expression, whereas the Tulahuen strain also induced overexpression of IL-11 (1.7-fold), IL-21 (9.4-fold), and colony stimulating factor 3 receptor (2.3-fold). The Y strain also led to increased expression of IL-21 transcript (4.4-fold). Concerning the membrane receptors that trigger JAK/STAT signaling, we found that the Brazil and Tulahuen strains both induced upregulation of prolactin receptor (Prlr, 3.5- and 3.9-fold, respectively). Cells infected with the CL strain showed increased expression of IL-3 (1.9-fold) and IL-21 (3.0-fold) receptors, as well as IFN-α receptor 1 (IfnaR1, 1.8-fold). The Y strain also increased the expression of IL-21 receptor (2.5-fold). Other constituents of this pathway were altered by the Y and CL strains: PIAS4 was upregulated by 1. 6- and 2.0-fold, respectively. Conversely, the Tulahuen strain downregulated expression of *protein inhibitor of STAT1 and STAT3* (PIAS1 and PIAS3) by 1.7- and 2.5-fold, respectively (Figure 2).

### Distinct Modulation in Cell Cycle Pathway by *Trypanosoma cruzi* Strains

**Figure 3:**
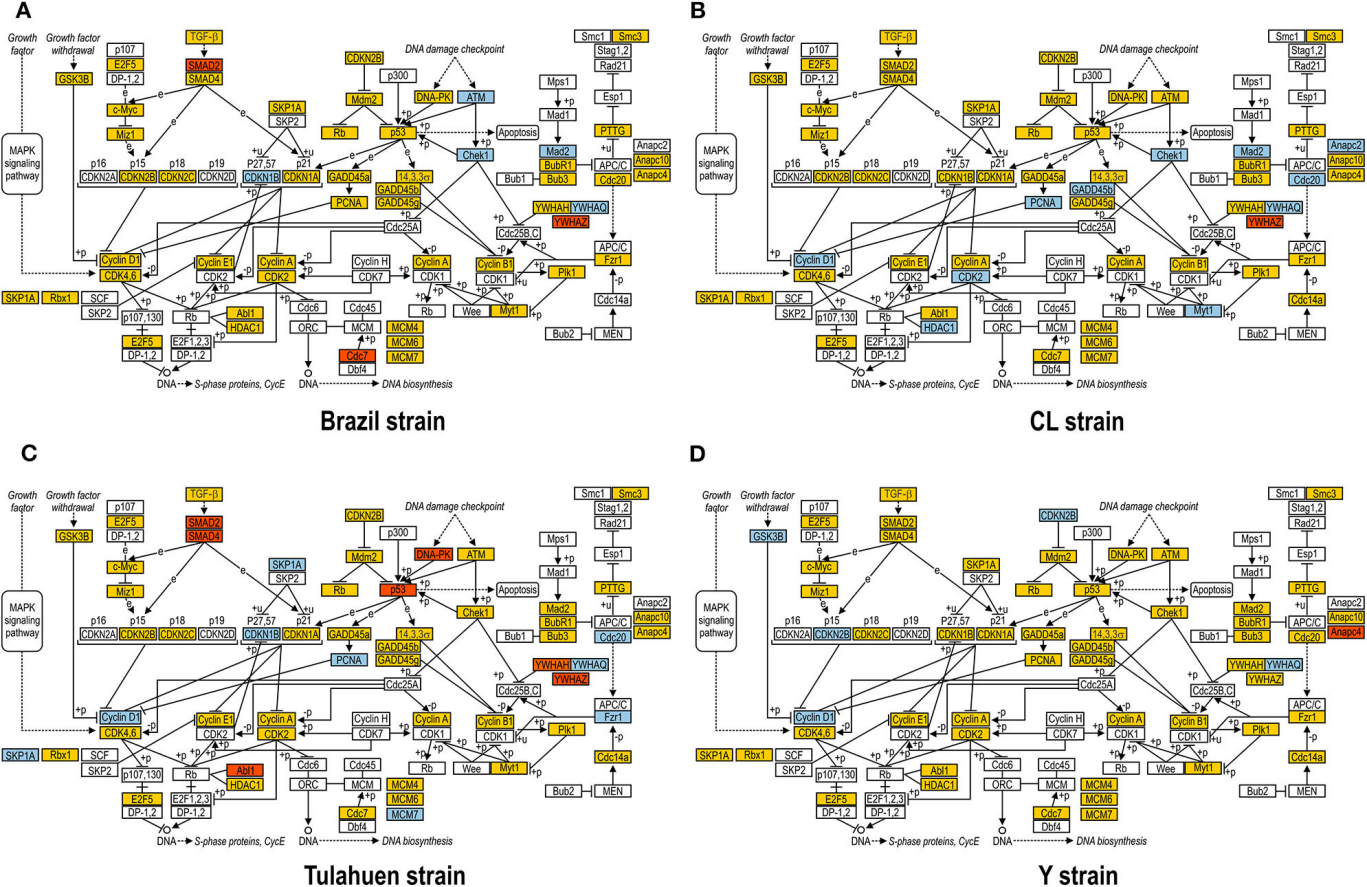
Kyoto Encyclopedia of Genes and Genomes (KEGG) analysis of the Cell Cycle Pathway in *Trypanosoma cruzi*-infected myoblasts. The Cell Cycle Pathway obtained from the KEGG platform was used as template to highlight the effect of Brazil **(A)**, CL **(B)**, Tulahuen **(C)**, and Y **(D)** strain in L6E9 rat myoblasts. Blue boxes indicate genes that were significantly upregulated (>1.5-fold), and red boxes indicate those that were significantly downregulated (<-1.5-fold). Yellow boxes indicate genes that showed no significant alteration in infected vs. control cultures, and white boxes indicate genes that were absent in the analysis (i.e., those that for any reason did not match the exclusion criteria; e.g., a positive signal in all four biological replicas of each group). Distinct stimuli can regulate the expression of cyclins and cyclin-dependent kinases (CDKs) and transcription factors (E2F5, and E2F1, 2, and 3) that coordinate cell cycle progression, DNA biosynthesis, and S-phase protein synthesis.

JAK/STAT pathway activation may lead to changes in cell cycle components, and one of these (*Tyrosine 3-Monooxygenase/Tryptophan 5-Monooxygenase Activation Protein Theta:* Ywhaq) was one of the few genes found to be upregulated by all four strains studied (Adesse et al., 2010a). In addition, three out of the four strains used in this work led to increased expression of *Cyclin D1* (Ccnd1), a major player in the cell cycle pathway, as has also been previously reported (Bouzahzah et al., 2008). Thus, we investigated how *T. cruzi* altered the cell cycle pathway by using KEGG templates (Figure 3), and the findings are described below.

The cell cycle pathway was most altered by the CL and Tulahuen strains showing 22% (12 of 55 analyzed spots) and 27% (15/55 spots) altered genes, respectively. The Brazil strain (TcI) and Y strain (isolated from São Paulo state, Brazil, TcII) had an impact on these two pathways, although to a lesser extent, affecting eight and five of the 55 measured genes, respectively. Specific Cell Cycle Pathway genes whose expression was altered following infection are described below.

In the Brazil strain, upregulated genes included *cyclin-dependent kinase inhibitor 1B* (*Cdkn1b*, 2.3-fold); *ATM serine/threonine kinase* (ATM, 2.9-fold); *checkpoint kinase 1 homolog* (*Chek1*, 2.3-fold), and *MAD2* (*mitotic arrest deficient, homolog)-like 1* (*Mad2l1*, 1.8-fold). Downregulated genes includes *SMAD family member 2* (SMAD2, −1.5-fold) and *cell division cycle 7* (*Cdc7*, −1.8-fold). Whereas the theta polypeptide of tyrosine 3-monooxygenase/tryptophan 5-monooxygenase activation protein (*Ywhaq*) was upregulated (2.9-fold), the zeta polypeptide (*Ywhaz*) was downregulated (−1.9-fold) (Figure 3A).

In the CL strain, upregulated genes included *Cyclin D1* (*CycD*, 2.0-fold), *histone deacetylase 1* (HDAC1, 1.6-fold), *cyclin dependent kinase 2* (*Cdk2*, 2.0-fold), *proliferating cell nuclear antigen* (PCNA, 1.8-fold), *growth arrest and DNA-damage-inducible beta* (*Gadd45b*, 2.1-fold), Checkpoint kinase 1 homolog (Chek1, 3.5-fold), *protein kinase, membrane associated tyrosine/threonine 1* (*Pkmyt1*, 1.8-fold), *MAD2* (*mitotic arrest deficient, homolog)-like 1* (*yeast)* (*Mad2l1*, 1.9-fold), *anaphase promoting complex subunit 2* (*Anapc2*, 1.8-fold), and *cell division cycle 20* (*Cdc20*, 2.9-fold). Regarding the members of the 14-3-3 complex, CL infection upregulated the *theta polypeptide* (*Ywhaq*, 2.9-fold) (Figure 3B).

The Tulahuen strain was very disruptive for cell cycle genes, with 27% significantly altered. Upregulated genes included *S-phase kinase-associated protein 1* (*Skp1a*, 2-fold), *CyclinD1* (Ccnd1, 2-fold), cyclin-dependent kinase inhibitor 1B (*Cdkn1b*, 1.7-fold), minichromosome maintenance deficient 2 mitotin (MCM2, 1.5-fold), *fizzy/cell division cycle 20 related 1* (*Fzr1*, 2.3-fold), *cell division cycle 20 homolog* (Cdc20, 1.8-fold), *proliferating cell nuclear antigen* (*PCNA*, 1.6-fold). Downregulated genes included MAD homolog 2 (SMAD2, −1.9-fold), MAD homolog 4 (SMAD4, −1.7-fold), V-abl Abelson murine leukemia viral oncogene homolog 1 (*Abl1*, −3.3-fold), *protein kinase, DNA activated, catalytic polypeptide* (*Prkdc*, −1.8-fold), and *tumor protein p53* (tp53, −1.9-fold). Three members of the *tyrosine 3-monooxygenase/tryptophan 5-monooxygenase activation protein* were affected by the Tulahuen strain infection: downregulated the *eta* (*Ywhah*, −*1.8-fold*) and *zeta* (*Ywhaz*, −*1.8-fold*), and upregulated the *theta* (*Ywhaq*, 1.8-fold) (Figure 3C).

The Y strain was the least disruptive for the cell cycle pathway, leading to upregulation of *glycogen synthase kinase 3 beta* (*Gsk3b* 1.5-fold), *cyclin-dependent kinase inhibitor 2B* (*Cdkn2b*, 1.7-fold), *Ywhaq* (2.6-fold), and downregulation of *anaphase promoting complex subunit 4* (*Anapc4*, −1.6-fold) (Figure 3D).

### Expression Coordination

The pathway expression analysis shown in Figures 2, 3 provides information on whether genes within a pathway are individually affected by a treatment or condition but does not indicate whether expression of genes within a pathway is coordinately expressed. To examine this issue, we used pairwise Pearson coefficients to determine whether expression differences in individual samples were correlated with one another, possibly indicating that the encoded proteins may be functionally interlinked (Spray and Iacobas, 2007, Figure 4).

**Figure 4:**
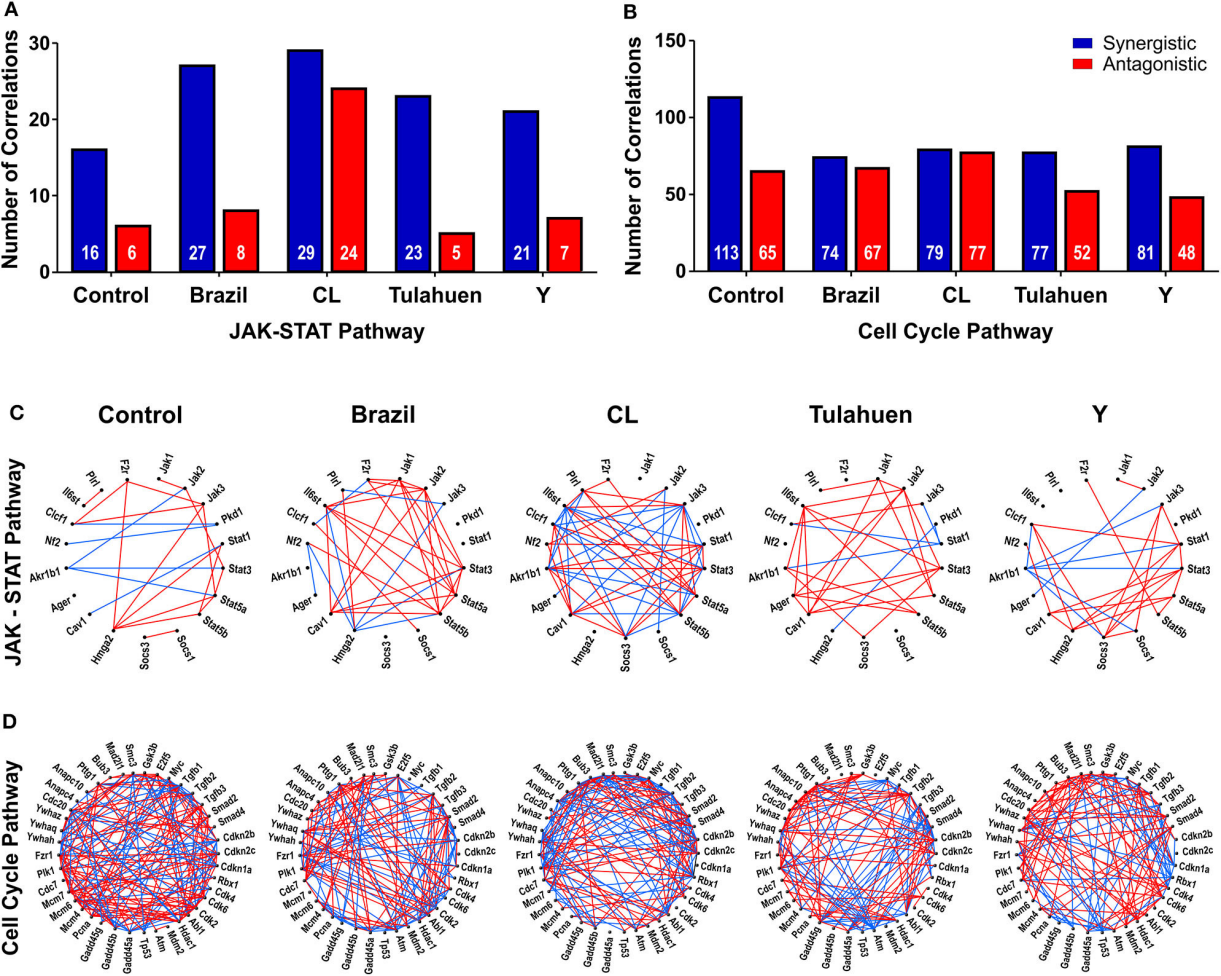
*Trypanosoma cruzi* infection affects the coordination of genes belonging to JAK/STAT and cell cycle pathways. The number of synergisms (blue bars) and antagonisms (red bars) for the JAK/STAT **(A)** and Cell Cycle **(B)** pathways are shown, as determined by Pearson correlation analysis. The circles in **(C,D)** depict the synergistic (blue lines) and antagonistic (red lines) correlations between genes of each pathway, in uninfected and *T. cruzi*-infected datasets.

From these measurements, we determined the number of gene pairs with significant pairwise Pearson correlation coefficients, with synergistic correlations shown in blue and antagonistic in red in Figures 4A,B. Graphical representation of the coordination interactions (synergistic and antagonistic) of genes in the JAK/STAT and Cell Cycle pathways are shown in Figures 4C,D, respectively.

In the non-infected control group, the JAK/STAT signaling pathway had 16 synergistic and six antagonistic coordination. Samples obtained for each of the four strains exhibited higher numbers of synergisms (27, 29, 23, and 21 for Brazil, CL, Tulahuen, and Y, respectively); antagonistic correlations for the Brazil, Talahuen, and Y strains were similar to those of control (six antagonisms each) (Figure 4A). The CL strain induced a much higher number of antagonisms (24) (Figures 4A,C).

In the Cell Cycle Pathway, the control group showed 113 synergisms and 65 antagonisms. In contrast to what was observed in gene coordination in the JAK/STAT signaling pathway, *T. cruzi* infection by all strains resulted in fewer synergisms (ranging from 74 to 81, Figures 4B–D).

**Figure 5:**
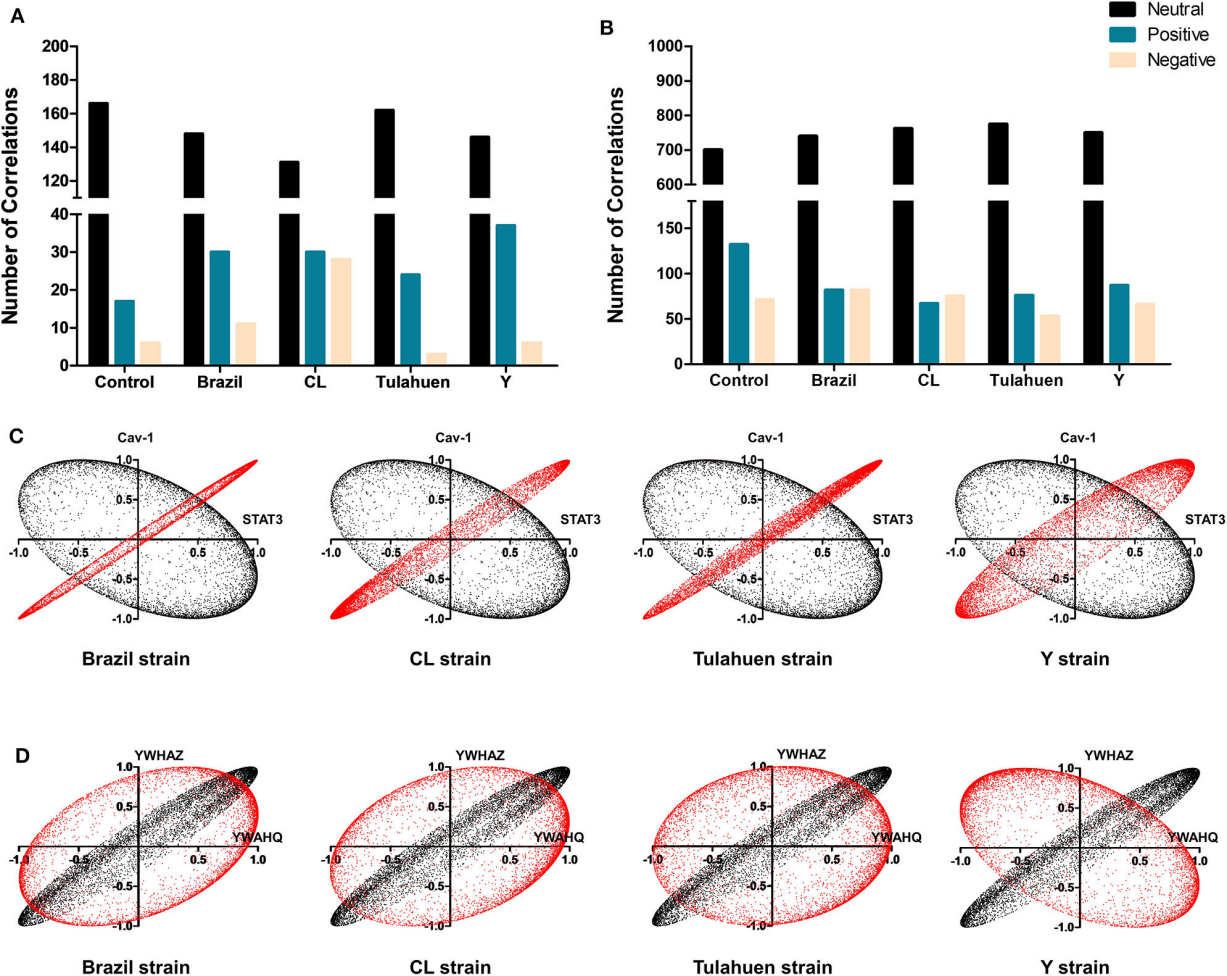
Differential effects of infection on global gene correlation. Plots of correlation coefficients between the expression levels of the indicated genes with each other genes differentially expressed in each experimental condition. The number of neutral (black bars), positive (blue bars), and negative correlations (yellow bars) was quantified among genes belonging to the JAK/STAT **(A)** and Cell Cycle **(B)** pathways*. T. cruzi* infection increased the number of positive correlations of the JAK/STAT pathway, whereas an opposite effect was observed for Cell Cycle genes. In **(C,D)** are depicted representative correlation profiles of each pathway: *cav-1* and *stat3* of the JAK/STAT pathway **(C)** and *YWHAZ* and *YWHAQ* of the Cell Cycle Pathway **(D)**. Profiles of the uninfected controls are represented by the black dots and *T. cruzi*-infected by red dots.

Additionally, we determined whether infection by each of the *T. cruzi* strains altered the number of correlations of the JAK/STAT (Figure 5A) and Cell Cycle genes (Figure 5B) with all other genes quantified on the arrays. Overall, the number of neutral correlations was fairly constant for genes in both pathways, and negative correlations were similar except for a larger number in the CL genes in the JAK/STAT pathway. By contrast, the number of positive associations was higher in all infected than uninfected groups for JAK/STAT and lower in all infected groups for the Cell Cycle Pathway. To illustrate the effect that *T. cruzi* infection had on coordination between members of the JAK/STAT pathway, we show in Figure 5C the coordination profile between STAT3 and caveolin-1. In the control condition (shown in black), these two genes had a neutral profile (corresponding to a broad ellipse covering all quadrants of the graph), which was altered to a variably narrow but significantly positive profile in each of the infected conditions (shown in red). An example of conversion of a coordination of Cell Cycle genes from a positive coordination under control conditions to a neutral profile in infected cells is shown in Figure 5D. Uninfected cultures displayed a positive profile (black dots) for the *Ywaq* and *Ywhaz* pair of genes. In infected samples from each strain (shown in red), the coordination changed to a neutral profile.

## Discussion

*Trypanosoma cruzi* infection results in CD that has different clinical forms that include asymptomatic, cardiac, digestive, and neurological features (WHO, 2019). These diverse outcomes may be related to the environment, host, and parasite genetic variability (Lewis et al., 2016) and by a combination of all these variables. *T. cruzi* genetic isolates are currently divided into six DTUs according to genetic, biochemical, and/or biological markers (Zingales et al., 2009); and strains from TcII, TcV, or TcVI were associated with chronic infection (Zingales, 2018). Here, we reanalyzed data from a previously published transcriptomic profiling of the infection of rat myoblasts by four *T. cruzi* strains, in order to further understand the impact that infection has on JAK/STAT signaling and cell cycle pathways and to understand the different outcomes of infection.

One of the few genes showing increased expression increased by all four strains of *T. cruzi* was *Clcf1* (Adesse et al., 2010a). CLCF1 belongs to the IL-6 cytokine family that includes IL-6, IL-11, ciliary neurotrophic factor (CNTF), leukemia inhibitory factor (LIF), oncostatin M (OSM), cardiotrophin 1 (CT-1), and IL-27.

Cytokines and growth factors commonly mediate their actions through the JAK/STAT pathway, which is associated with such cellular functions as inflammation, apoptosis, and cell-cycle control (Barry et al., 2007). IL-6 and IL-10 induce STAT3 activity (Barry et al., 2007). In Tulahuen strain infection, IL-6/pSTAT3 protects cardiomyocytes through upregulation of anti-apoptotic factor Bcl-2 (Ponce et al., 2012), thus maintaining the survival of the host cell that is beneficial for parasite persistence. IL-10/STAT3 signaling induces the SOCS-3 gene reducing tissue damage inducers such as pro-inflammatory factors nitric oxide synthase (NOS2) and tumor necrosis factor (TNF)-α in *T. cruzi* RA strain-infected cardiomyocytes culture (Hovsepian et al., 2013).

IFNs activate predominantly STAT1 and STAT2 (Barry et al., 2007). IFNγ/STAT1 signaling protected fibroblasts against CL Brener and Y infection by inhibition of amastigote growth (Stahl et al., 2014) that could explain the higher *T. cruzi* (Brazil strain) replication and dissemination in STAT1 knockout mice (Kulkarni et al., 2015). Interestingly, infection of STAT6- but not STAT4-knockout mice with this same (Brazil) strain resulted in decreased parasitemia, inflammation, and mortality when compared with wild-type mice (Tarleton et al., 2000). Therefore, different ways of modulating this pathway may induce different clinical aspects of the infection.

Our results showed that three out of four isolates of *T. cruzi* that were tested had nodules of predicted PPI of Cell Cycle process within their upregulated genes and that one (Brazil) had such interactions among the downregulated genes. Regarding the pairwise coordination profiling of genes belonging to the cell cycle pathway, we verified an overall reduction of synergistic interactions induced by all four strains. Accordingly, infection of L6E9 myoblasts by the Brazil strain led to no significant alteration of cyclin D1 promotor activity or cyclin D1 protein stability in infected cultures (Bouzahzah et al., 2008).

It is well-documented that *T. cruzi* affects host cell proliferation (Bouzahzah et al., 2006; Droguett et al., 2017; Duran-Rehbein et al., 2017). Curiously, one work that utilized the Y strain of *T. cruzi* showed that late mitotic genes were downregulated in infected cultures of vascular smooth muscle cells and fibroblasts (Costales et al., 2009), indicating defects in cytokinesis. This finding reinforces the fact that host cell or even host animal background plays a complimentary role on the course of infection. This seems to be the case of vascular smooth cells that were shown to have increased proliferation when infected by the Tulahuen strain of this parasite (Hassan et al., 2006).

Interestingly, our study showed that infected cultures had a decrease in the number of neutral correlations in JAK/STAT-related genes and an increase in the number of positive profiles. We exemplified this phenomenon with the correlation of caveolin-1 and STAT3. Caveolins are implicated in transcytosis of macromolecules, cholesterol transport, and signal transduction (Li et al., 2005). Knockout mice for caveolin-1, caveolin-2, and caveolin-3 develop hypertrophic cardiomyopathy with increase in fibrosis (Park et al., 2002; Cohen et al., 2003; Augustus et al., 2008). Experimental CD in mice also affects caveolins (Adesse et al., 2010b), with subsequent activation of MAPK signaling pathways (Huang et al., 2003), thus leading to remodeling of heart tissue.

The tyrosine 3-monooxygenase/tryptophan 5-monooxygenase activation proteins are a family of molecular chaperones commonly referred to as 14-3-3 proteins. The family consists of seven transcripts in mammals: 14-3-3β (YWHAB), 14-3-3γ (YWHAG), 14-3-3ε (YWHAE), 14-3-3ζ (YWHAZ), 14-3-3η (YWHAH), 14-3-3θ (YWHAQ), and 14-3-3σ also known as stratifin (SFN) (MacKay et al., 2011). YWHAH for instance has a well-established cardioprotective role in cases of cardiac overload. Mice with a dominant mutation in 14-3-3 proteins display reduced survival, left ventricular fraction, and fraction shortening (Thandavarayan et al., 2011; Sreedhar et al., 2016). The fact that 14-3-3 transcripts are altered in myoblasts infected with *T. cruzi* reinforces the idea that 14-3-3 proteins may contribute to cardiomyocyte apoptosis, inflammation, fibrosis, and hypertrophy observed in cardiac forms of Chagas chronic disease.

In summary, the bioinformatic tools used in this work allowed the further description of the differential impact of *T. cruzi* genetic background on host cell transcriptome, as a good predictor of biological outcomes. Although *Clcf-1* and *Ywhaq* were equally altered in the infected L6E9 cells, their network ensemble was in fact composed of different transcripts, which may lead to variations in the degrees of activations in these molecular pathways. These observations are important to deepen the understanding of how CD can present multiple pathologies, according to parasite background, combined with host diversity and environmental aspects. Such variability should be taken in consideration when proposing chemotherapeutic or immunomodulatory approaches to control of this disease.

## Data Availability Statement

The raw datasets generated in this study can be found in NCBI, accession number GSE18175.

## Author Contributions

DA, LG, DS, HT, and DI conceptualized the study. DA, DI, and DS contributed to methodology, resources, and funding acquisition. PCV, PHV, LN, TM, and LC helped with the validation. SI, DI, and TM did the formal analysis. DA, LN, PCV, PHV, LC, and TM carried out the investigation. DA and LN contributed to data curation and writing the original draft. DA, LG, DS, and DI wrote, reviewed, and edited the manuscript. DA was responsible for the visualization and project administration. DA, DI, DS, and HT supervised the study. All authors contributed to the article and approved the submitted version.

## Conflict of Interest

The authors declare that the research was conducted in the absence of any commercial or financial relationships that could be construed as a potential conflict of interest.

## Supplementary Material

Supplementary Figure 1 Examples of genes synergistically (*Antxr1* = ANTXR cell adhesion molecule 1), antagonistically (*Dus3l* = dihydrouridine synthase 3-like), and independently (*Golim4* = golgi integral membrane protein 4) expressed genes with *Clcf1* (cardiotrophin-like cytokine factor 1) in control L6E9 rat myoblasts. Numbers in brackets are the Pearson correlation coefficients.
