# Supplementary figures and images for: Trypanosoma cruzi Promotes Transcriptomic Remodeling of the JAK/STAT Signaling and Cell Cycle Pathways in Myoblasts
Source: Front Cell Infect Microbiol. 2020 Jun 17;10:255. doi: 10.3389/fcimb.2020.00255 (PMC7313395; doi:10.3389/fcimb.2020.00255)

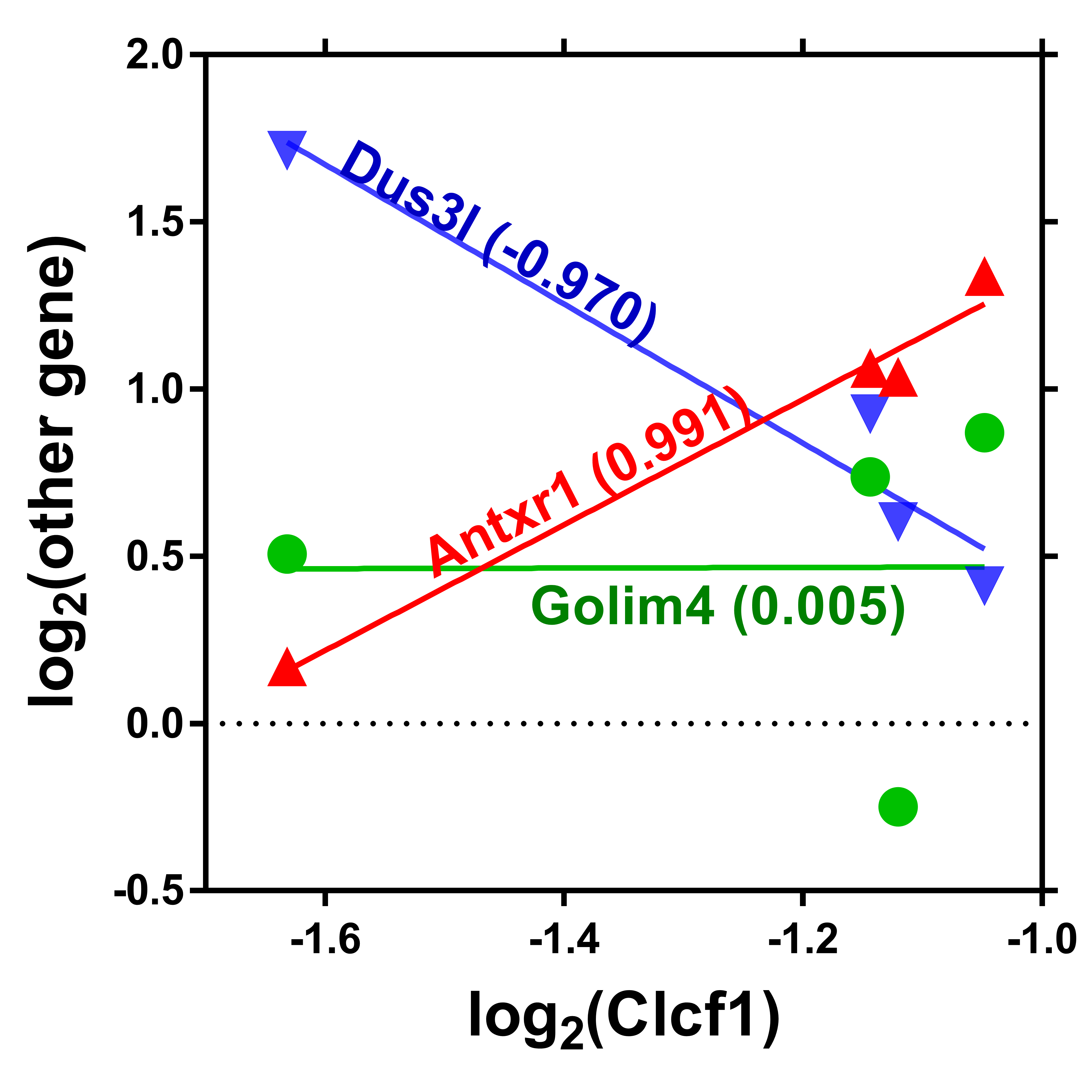

Supplement: Supplementary Figure 1 — Examples of genes synergistically (Antxr1 = ANTXR cell adhesion molecule 1), antagonistically (Dus3l = dihydrouridine synthase 3-like), and independently (Golim4 = golgi integral membrane protein 4) expressed genes with Clcf1 (cardiotrophin-like cytokine factor 1) in control L6E9 rat myoblasts. Numbers in brackets are the Pearson correlation coefficients. [file Image_1.TIF]
